# Supplementary material for: Metabolic reprograming mediated by tumor cell-intrinsic type I IFN signaling is required for CD47-SIRPα blockade efficacy
Source: Nat Commun. 2024 Jul 9;15:5759. doi: 10.1038/s41467-024-50136-z (PMC11233683; doi:10.1038/s41467-024-50136-z)
Supplement: Supplementary file 1 — Supplementary Information [file 41467_2024_50136_MOESM1_ESM.pdf]

Supplementary Information

for

Metabolic reprograming mediated by tumor cell-intrinsic type I IFN signaling is required for CD47-SIRPα blockade efficacy

Supplementary Figures 1-9

Supplementary Tables 1-3

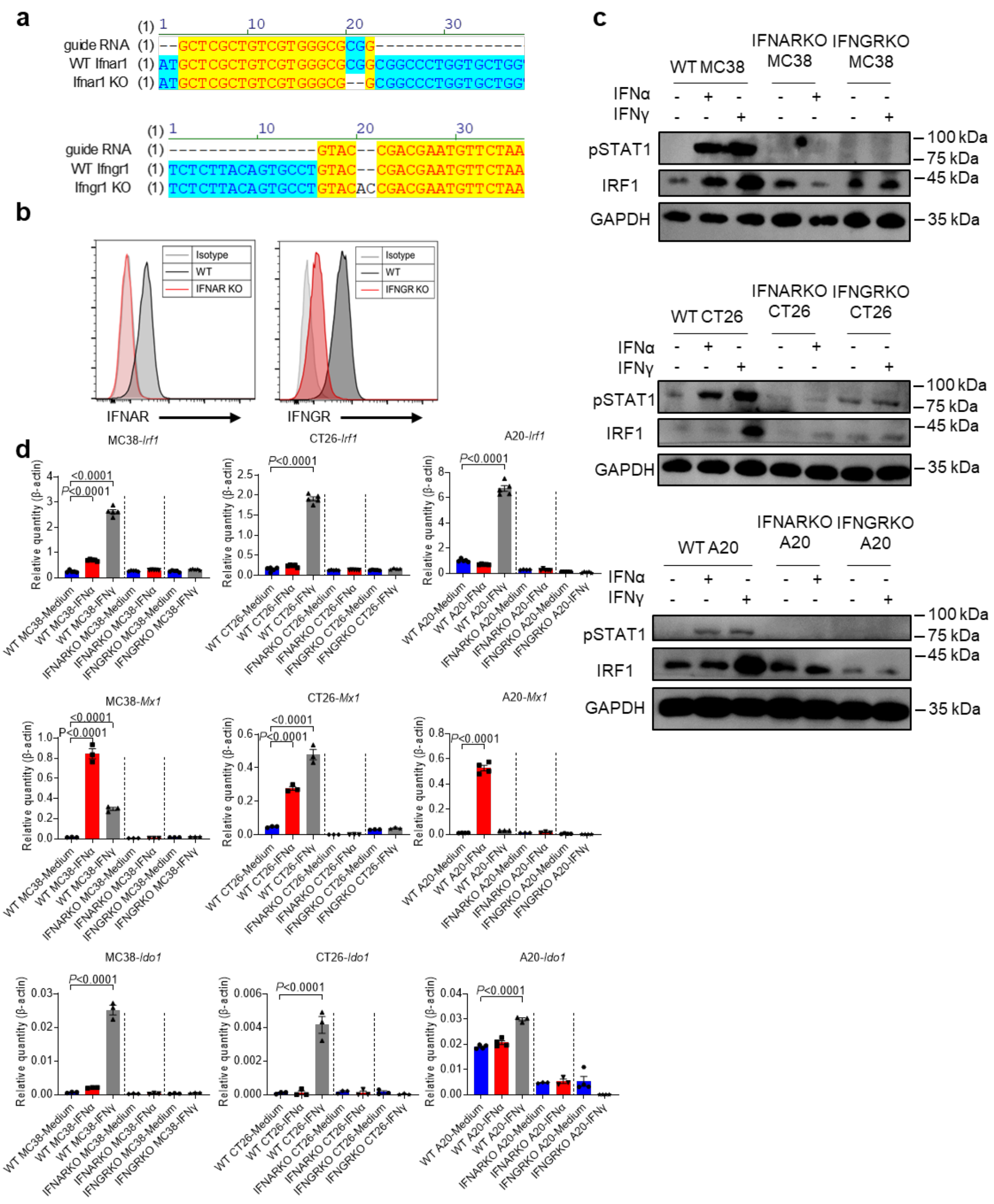

Supplementary Figure 1. The construction of IFNAR1 KO and IFNGR1 KO tumor cell lines. IFNAR1 and IFNGR1 KO MC38 clones were confirmed by genomic sequencing **(a)** and FACS staining **(b)**. **c** The cells as indicated were stimulated by 1 µg/mL recombinant IFN-α or 100 ng/ml recombinant IFN-γ for 48 h, and the expression of pSTAT1 and IRF1 was determined by Western blot. **d** As in **(c)**, the expression of *Irf1*, *Mx1* and *Ido1* were measured by real-time PCR (n=3-5 biologically independent samples). Data are representative of three independent experiments in b, two independent experiments in c, d. One-way ANOVA and multiple comparisons test was used in d. Data are presented as mean values ± SEM. Source data are provided as a Source Data file.

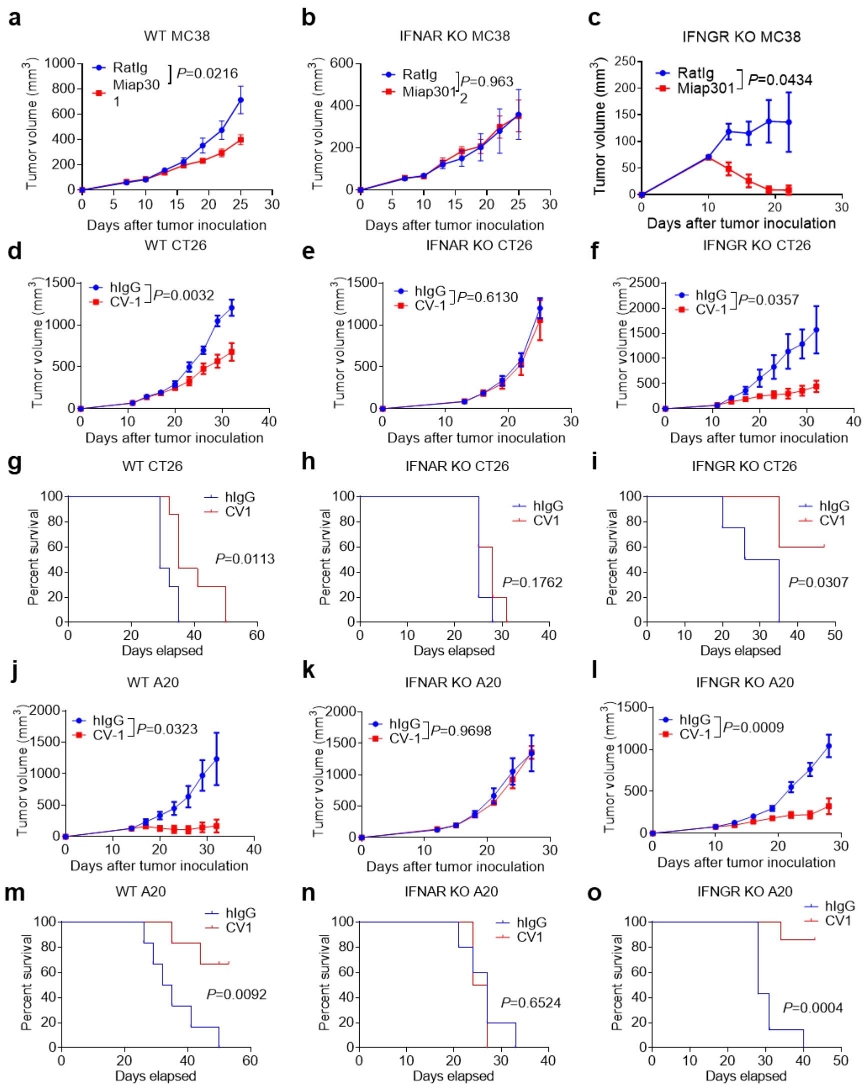

Supplementary Figure 2. Tumor cell-intrinsic IFN-I signaling is essential for CD47 blockade therapy in mice.

a-c C57BL/6 mice bearing tumors of WT MC38 (n=6 mice per group) **(a)**, IFNAR1 KO MC8 (n=6 mice per group) **(b)** or IFNGR1 KO MC38 (n=7 mice per group) **(c)** were treated i.t. with Miap301 or RatIg every three days. Tumor volume was measured at indicated time. C57BL/6 mice bearing tumors of WT CT26 (n=7 mice per group) **(d, g)**, IFNAR1 KO CT26 (n=5 mice per group) **(e, h)**, IFNGR1 KO CT26 (n=4 mice in hIgG group and n=5 in CV1 group) **(f, i)**, WT A20 (n=6 mice per group) **(j, m)**, IFNAR1 KO A20 (n=5 mice in hIgG group and n=4 in CV1 group) **(k, n)** or IFNGR1 KO A20 (n=7 mice per group) **(l, o)** were treated i.t. with CV-1 or hIgG every three days. Tumor volume was measured at indicated time. The survival curves were shown, Data are representative of two independent experiments in a-c, three independent experiments in d-o. Two-tailed unpaired Student's t-test was used in a-f, j-l. Log-rank (Mantel-Cox) test was used in g-i, m-o. Data are presented as mean values ± SEM. Source data are provided as a Source Data file.

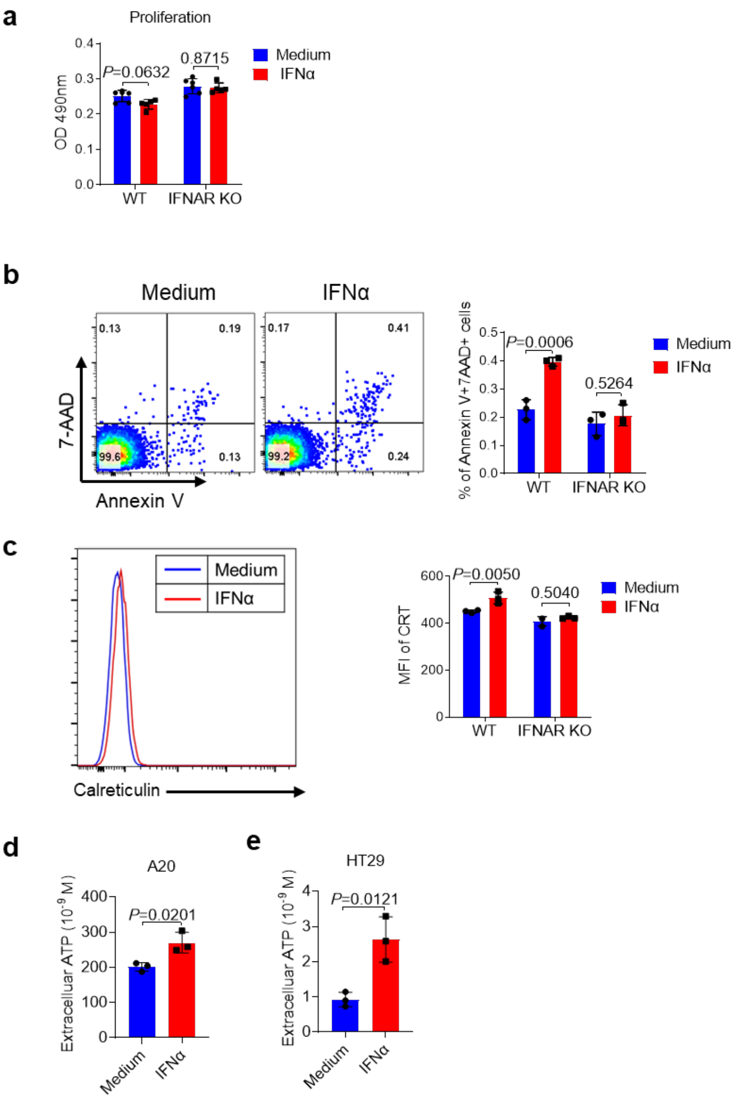

Supplementary Figure 3. IFN-I effects on cancer cell proliferation, apoptosis and DAMP production.

MC38 cells were treated by IFN-α for 48 hours. **a** The proliferation of cells was measured by CCK-8 assay (n=6 biologically independent samples in Medium group and n=5 biologically independent samples in IFNα group). **b** The apoptosis of MC38 cells was measured by FACS (n=3 biologically independent samples per group). **c** The exposure of CRT was measured by FACS (n=3 biologically independent samples per group). **d** A20 cells were treated by IFN-α for 48 hours. The concentration of extracellular ATP was measured (n=3 biologically independent samples per group). **e** HT29 cells were treated with IFN-α2b for 48 hours. The concentration of extracellular ATP was measured (n=3 biologically independent samples per group). Data are representative of three independent experiments in a-e. Two-way ANOVA and multiple comparisons test was used in a-c. Two-tailed unpaired Student's t-test was used in d, e. Data are presented as mean values ± SEM. Source data are provided as a Source Data file.

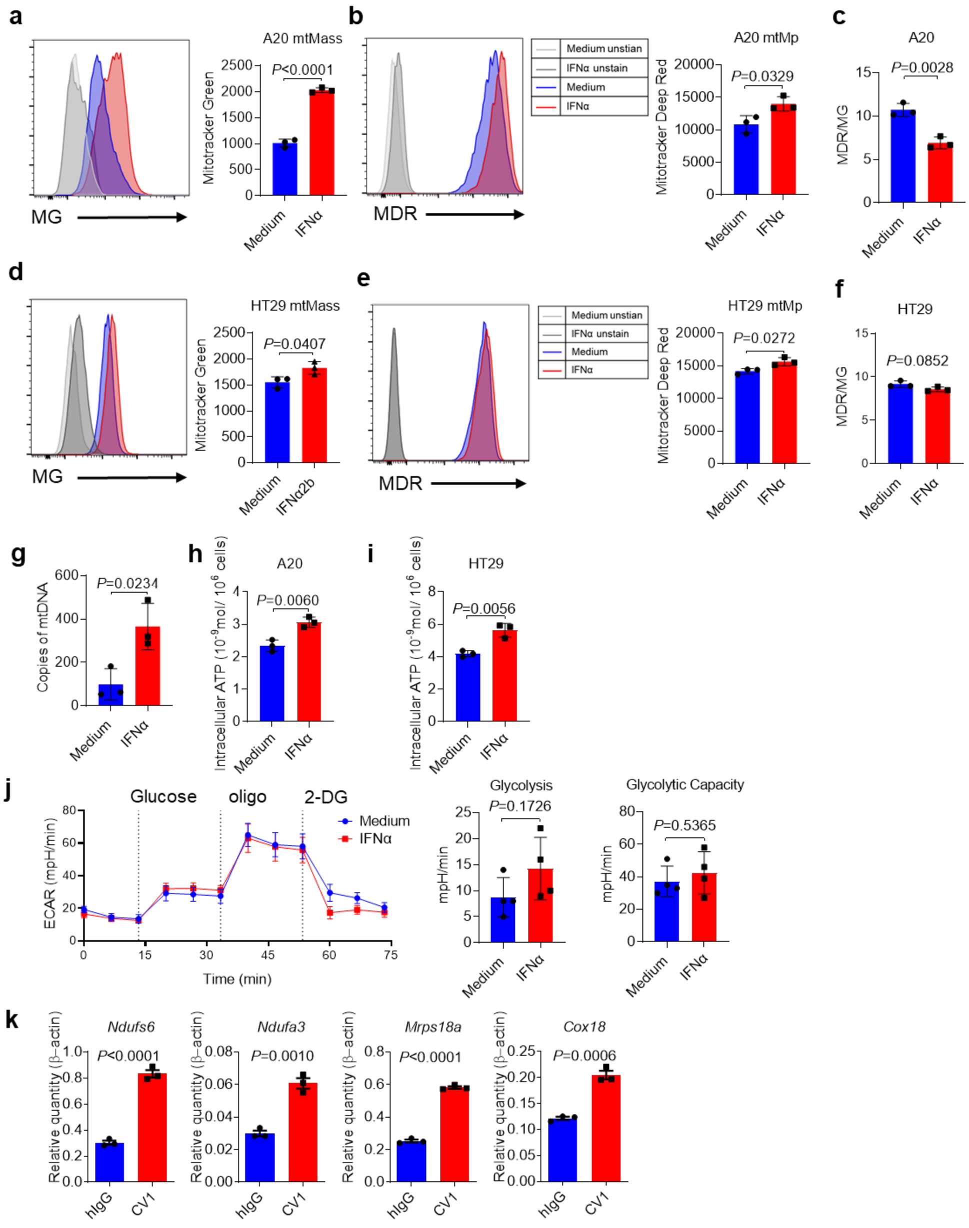

Supplementary Figure 4. IFN-I effects on mitochondria DNA, mitochondria OXPHOS and glycolysis.

A20 cells were treated with IFN-α for 48 hours. Mitochondrial mass **(a)** and membrane potential **(b)** were measured by FACS (n=3 biologically independent samples per group)**.** The ratio of MDR to MG was shown in **c.** HT29 cells were treated with IFN-α for 48 hours. Mitochondrial mass **(d)** and membrane potential **(e)** were measured by FACS (n=3 biologically independent samples per group)**.** The ratio of MDR to MG was shown in **f. g** MC38 cells were treated with IFN-α for 48 hours. Copy numbers of mitochondrial DNA were measured (n=3 biologically independent samples per group). A20 cells **(h)** and HT29 cells **(i)** were treated with IFN-α for 48 hours. Concentration of intracellular ATP was measured (n=3 biologically independent samples per group). **j** MC38 cells were treated with IFN-α for 48 hours. ECAR was measured by Seahorse assay (n=4 biologically independent samples per

group). **k** C57BL/6 mice bearing MC38 tumors were treated i.t. with CV-1 or hIgG every three days. Two days after the third treatment, CD45- cells were sorted. The expression of OXPHOS related genes were measured by real-time PCR (n=3 biologically independent samples per group). Data are representative of three independent experiments in a-i, k, two independent experiments in j. Two-tailed unpaired Student's t-test was used. Data are presented as mean values ± SEM. Source data are provided as a Source Data file.

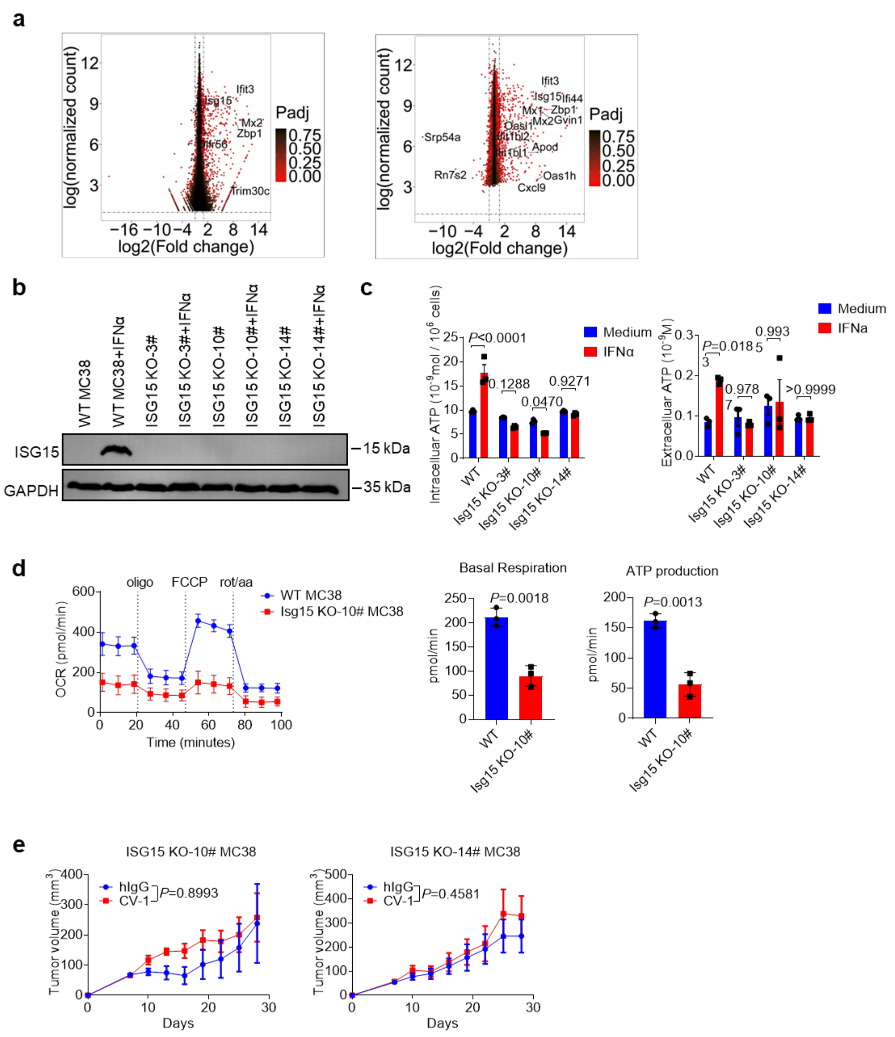

Supplemental Figure 5. IFN-Is promote tumor cell OXPHOS and ATP production via ISG15.

a RNA-seq analysis of WT MC38 cells stimulated with IFN-α for 6 h (left) and 24h (right). The differentially expressed genes between the medium and IFN-α treatment groups are shown in a volcano plot. (n=2 biologically independent samples in 6h-IFNa group and n=3 biologically independent samples per other group) **b** ISG15 KO MC38 clones were confirmed by Western blot. **c** WT, and three Isg15 KO MC38 cell clones (3#, 10# and 14#) were treated with IFN-α for 24 h. The concentration of intracellular ATP and extracellular ATP were measured (n=3 biologically independent samples per group). **d** OCR was measured by the Seahorse assay (n=3 biologically independent samples per group). **e** C57BL/6 mice (n=6 mice per group) bearing Isg15 KO MC38 tumors (10# or 14# clone) were treated with hIgG or CV-1 every three days. The tumor volume was measured at the indicated time. Data are representative of two independent experiments in a, b, d, three independent experiments in c, e. Two-way ANOVA and multiple comparisons test was used in c. Two-tailed unpaired Student's t-test was used in d, e. Data are presented as mean values ± SEM Source data are provided as a Source Data file.

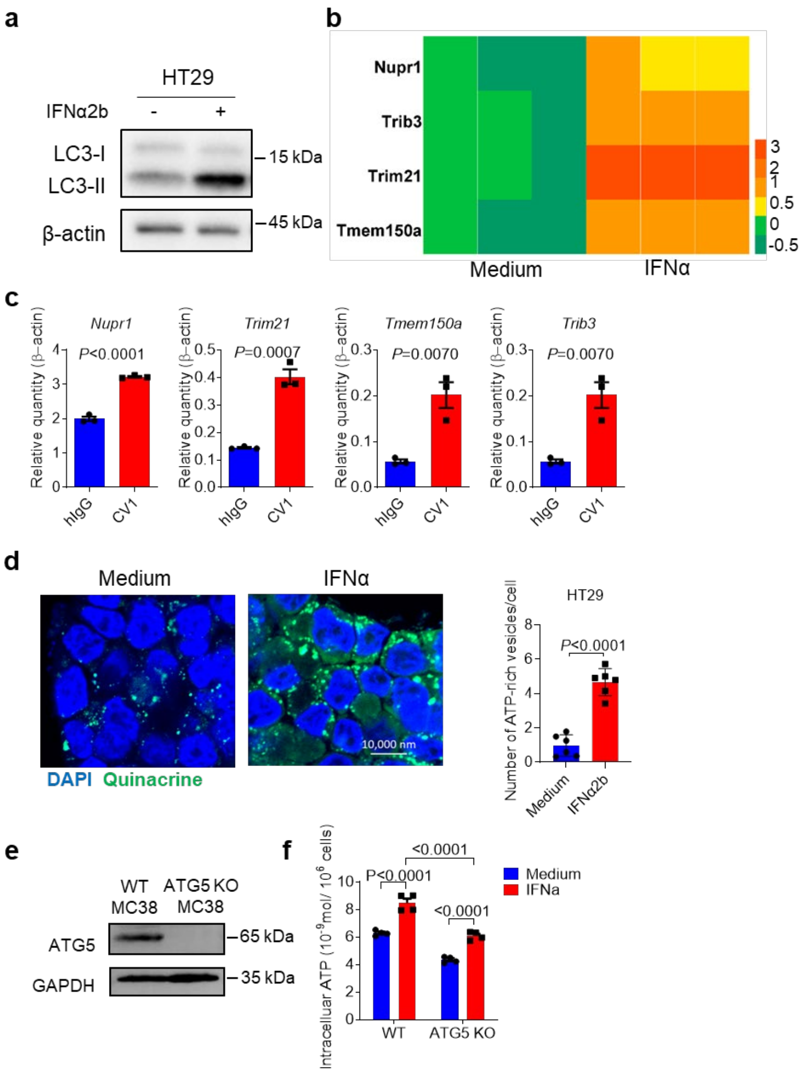

Supplemental Figure 6. Autophagy is regulated by IFN-I for ATP release.

a HT29 cells were treated with IFN-α2b for 48 h. Autophagy was measured by Western blot. **b** MC38 cells were treated with IFN-α for 48 h. The cells were harvested for RNA-seq. Heatmaps of IFN-α-stimulated genes that were also associated with autophagy regulation are shown (n=3 biologically independent samples per group). **c** C57BL/6 mice bearing MC38 tumors were treated i.t. with CV-1 or hIgG every three days. Two days after the third treatment, CD45- cells were sorted. The expression of autophagy-related genes was measured by real-time PCR (n=3 biologically independent samples per group). **d** ATP-rich vesicles of HT29 were measured by immunofluorescence staining (n=6 biologically independent samples per group). **e** ATG5 KO MC38 clones were confirmed by Western blot. **f** WT and Atg5 KO MC38 cells were treated with IFN-α for 48 hours. Intracellular ATP concentration was measured (n=4 biologically independent samples per group). Data are representative of two independent experiments in b, e, three independent experiments in a, c, d, f. Two-tailed unpaired Student's t-test were used in c, d. Two-way ANOVA and multiple comparisons test was used in f. Data are presented as mean values ± SEM. Source data are provided as a Source Data file.

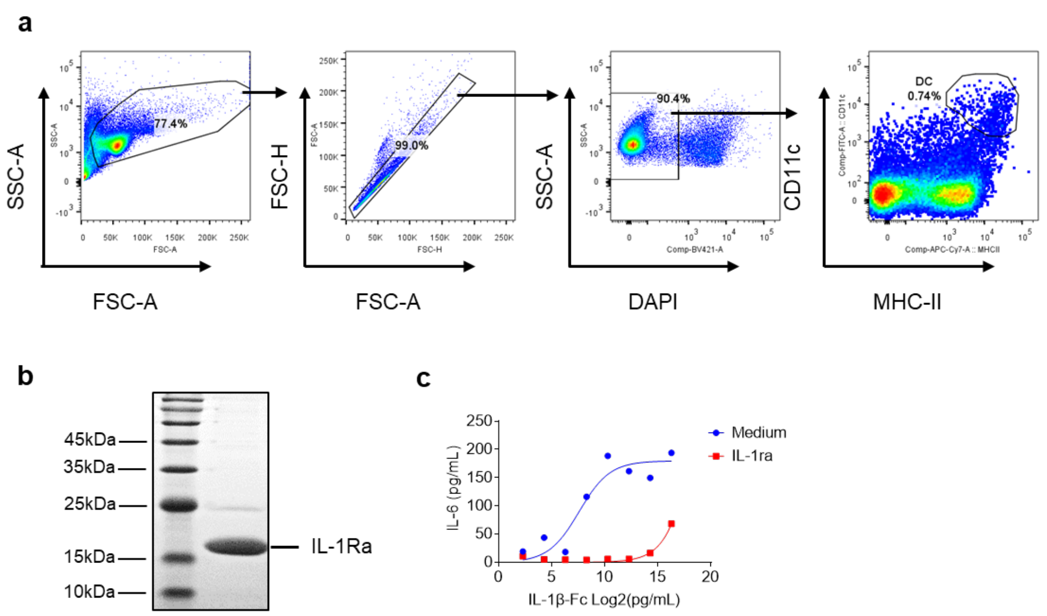

Supplemental Figure 7. Preparation and activity measurement of IL-1Ra, sequential gating strategies of DCs.

a The sequential gating strategies of DCs. **b** The expression of IL-1Ra was measured using SDS-PAGE. **c** The blocking efficiency of IL-1Ra to IL-1β was measured by the detection of IL-6 via CBA assay. Data are representative of two independent experiments in b, c. Source data are provided as a Source Data file.

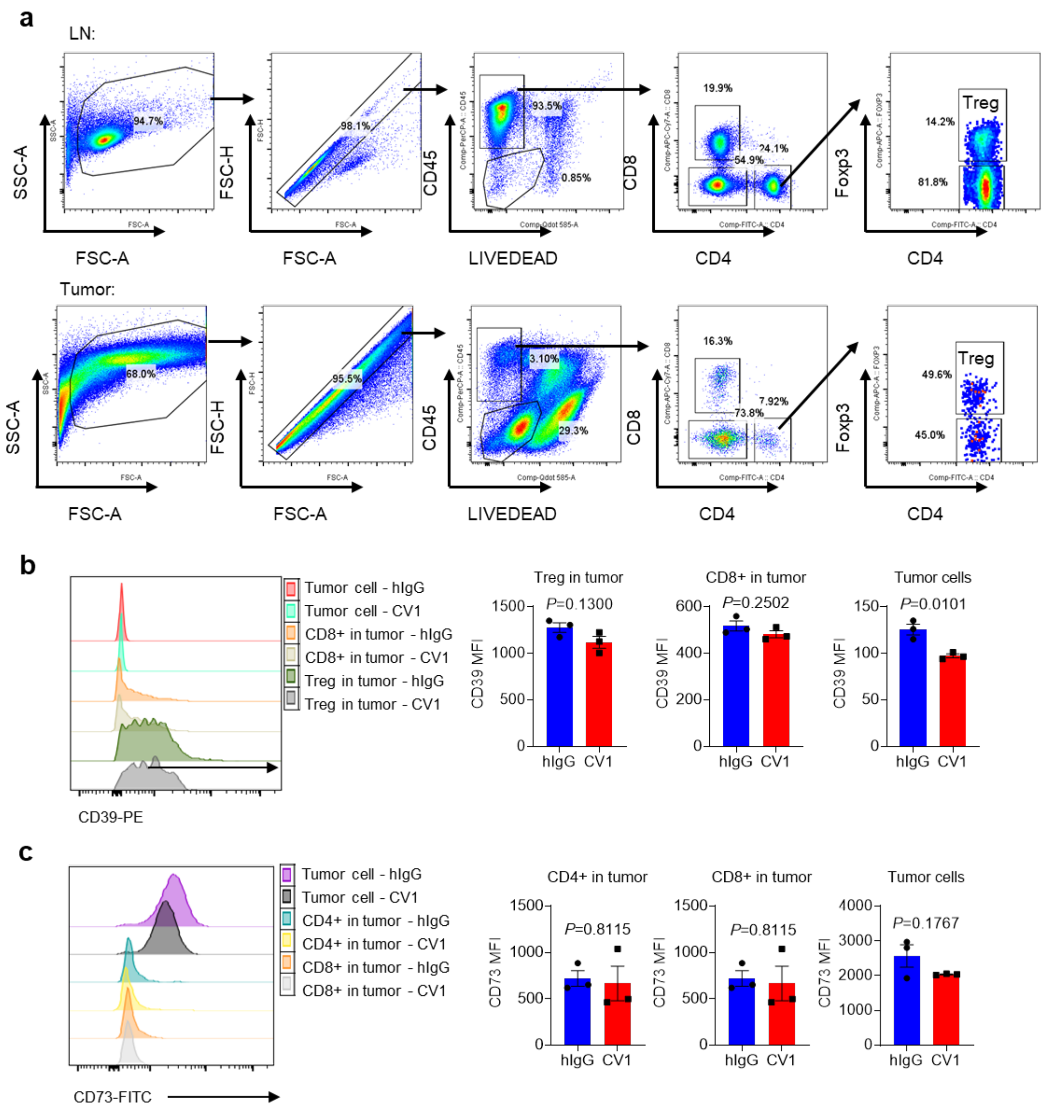

Supplemental Figure 8. CV-1 does not alter the expression of ATP-degrading enzymes.

a The sequential gating strategies of CD8+ T cells, CD4+ T cells and Treg cells in dLNs and tumors. C57BL/6 mice bearing WT MC38 tumors were treated i.t. with CV-1 or hIgG every three days. Two days after the third treatment, tumors were digested. **b** The expression of CD39 on Treg cells, CD8+ T cells and tumor cells was measured (n=3 mice per group). **c** The expression of CD73 on CD4+ T cells, CD8+ T cells and tumor cells was measured (n=3 mice per group). Data are representative of two independent experiments in b, c. Two-tailed unpaired Student's t-test were used. Data are presented as mean values ± SEM Source data are provided as a Source Data file.

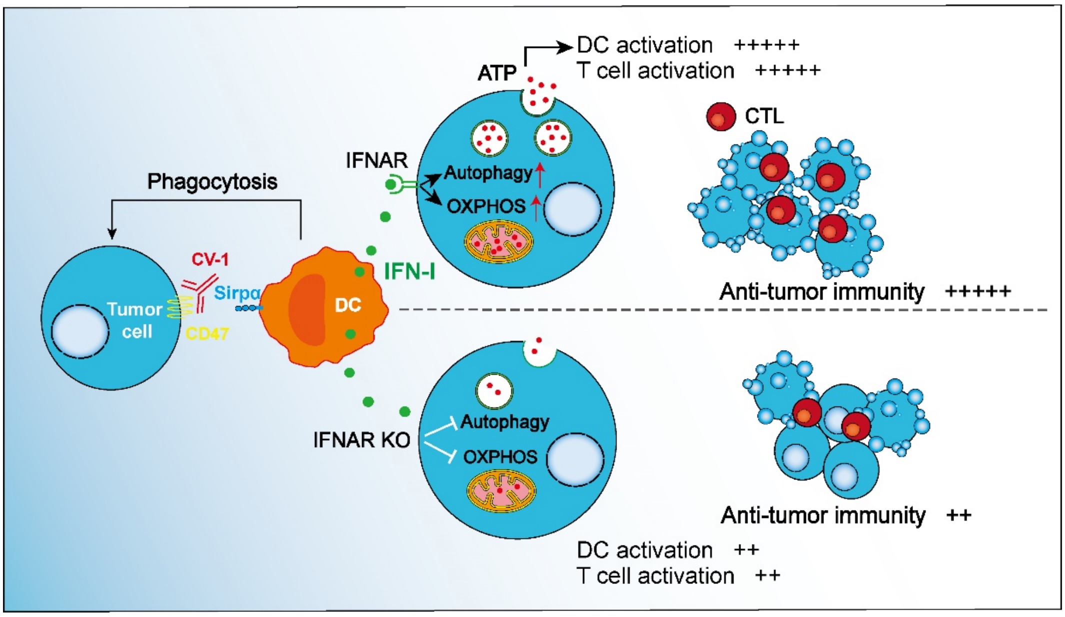

Supplemental Figure 9. Schema of metabolic reprograming mediated by tumor cell-intrinsic type I IFN signaling is required for CD47-SIRPα blockade efficacy.

Tumor cell-intrinsic type I IFN signaling is required for CD47-SIRPα blockade efficacy. Type I IFN reprograms tumor cell metabolism toward OXPHOS for ATP production. Type I IFN induces tumor cell autophagy for ATP extracellular release. Extracellular ATP enhances DC activation and T cell activation against tumors.

Supplementary Table 1: Guide RNA sequences used in this study.

Gene	Guide RNA (5’ to 3’)
Ifnar1	GCTCGCTGTCGTGGGCGCGG
Ifngr	ATTAGAACATTCGTCGGTAC
Atg5	AAGATGTGCTTCGAGATGTG
Ndufs6	CACGCATACCGGCCAGGTAA
Isg15	GCTGGGGGGTAACGATTTCC

Supplementary Table 2: Antibodies used in this study.

Reagent	Source	Clone/ Catalogue number	Conc.	Dilution
CD45 PerCP-5.5(Ly-5)	eBioscience	Clone: 30-F11; Cat. 45-0451	0.2 mg/ml	1:300
CD3e FITC	eBioscience	Clone: 145-2C11; Cat. 11-0031-82	0.5 mg/ml	1:300
CD19 PE	eBioscience	Clone: eBio1D3 (1D3); Cat. 12-0193-82	0.2 mg/ml	1:300
CD11c APC	Biolegend	Clone: N418; Cat. 117310	0.2 mg/ml	1:300
I-A/I-E (MHCII) APC/Cy7	Biolegend	Clone: M5/114.15.2; Cat. 107628	0.2 mg/ml	1:300
CD45.1 PE/Cy7	eBioscience	Clone: A20; Cat. 25-0453-81	0.2 mg/ml	1:300
CD44 APC/Cy7	Biolegend	Clone: IM7; Cat. 103028	0.2 mg/ml	1:300
CD4 PE/Cy7	eBioscience	Clone:GK1.5; Cat. 25-0041-82	0.2 mg/ml	1:300
CD8 APC-eFlourR 780	eBioscience	Clone: 53-6.7; Cat. 47-0081-82	0.2 mg/ml	1:300
Foxp3 APC	eBioscience	Clone: FJK-16s; Cat. 17-5773-82	0.2 mg/ml	1:100
CD80 PE	eBioscience	Clone: 16-10A1; Cat. 12-0801	0.2 mg/ml	1:300
CD39 PE	Biolegend	Clone: Duha59; Cat. 143803	0.2 mg/ml	1:300
CD73 biotin	Miltenyi	Clone: TY/11.8; Cat. 130-102-046	0.03mg/ml	1:10
CD47 APC	eBioscience	Clone: miap301; Cat. 17-0471-80	0.2 mg/ml	1:300
IFNγ PE	Biolegend	Clone: XMG1.2; Cat. 505808	0.2 mg/ml	1:100
TCRVα2 FITC	eBioscience	Clone: B20.1;	0.5 mg/ml	1:300

		Cat. 11-5812-82		
CD69 PE	eBioscience	Clone: H1.2F3; Cat. 12-0691	0.2 mg/ml	1:300
CD62L Alexa Fluor® 700	eBioscience	Clone: MEL-14; Cat. 56-0621-82	0.2 mg/ml	1:300
IFNAR-1 PE	Biolegend	Clone: MAR1-5A3; Cat. 127311	0.2 mg/ml	1:300
CD119 (IFN gamma Receptor 1) PE	eBioscience	Clone: 2E2; Cat. 12-1191-80	0.2 mg/ml	1:300
Phospho-STAT1 (Ser727) Polyclonal Antibody	Proteintech	Cat. 28977-1-AP	0.5 mg/ml	1:1000
IRF1 Polyclonal Antibody	Proteintech	Cat. 11335-1-AP	0.5 mg/ml	1:500
LC3	Sigma-Aldrich	Cat. L8918	1 mg/ml	1:1000
b-actin	Sigma-Aldrich	Clone: AC-15; Cat. A3854	2-4 mg/mL	1:50000
GAPDH	Sigma-Aldrich	Cat. G9545	1 mg/ml	1:200
Purified Anti-Mouse IFN-γ	BD Biosciences	Cat. 51-2525KC	1 mg/ml	1:200
Biotinylated anti-mouse IFN-γ	BD Biosciences	Cat. 51-1818KZ	0.5 mg/ml	1:250
streptavidin-HRP	BD Biosciences	Cat. 51-9000209	Not available	1:100

Supplementary Table 3: Primers used for real-time PCR in this study.

Primer	Sequence (5' to 3')
Isg15 F	TCTTTCTGACGCAGACTGTAGA
Isg15R	GGGGCTTTAGGCCATACTCC
Cox18 F	GATGGTCCAAGAGAGTGGCT
Cox18 R	AATGCCGTCTGAATGTGTGG
Ndufs6 F	GGGGAAAAGATCACGCATACC
Ndufs6 R	CAAAACGAACCCTCCTGTAGTC
Ndufa3 F	CAAAGCGAGTTCCCTCAGG
Ndufa3 R	ACACGCGATTTCCGCAGATAG
Mrps18a F	AATCGAGGAGTGCGTGAAGA
Mrps18a R	GCATCCTTCTGGAAGCTGTG
Nupr1 F	CCCTTCCCAGCAACCTCTAAA
Nupr1 R	TCTTGGTCCGACCTTTCCGA
Trim21 F	TCAGACCACCCTCCACATTC
Trim21 R	TGAAGAAAGGTCGCAGAGGT
Tmem150a F	CACCCTGGTCCTTAGTGGAG
Tmem150a R	CACCCTGGTCCTTAGTGGAG
Trib3 F	TGCAGGAAGAAACCGTTGGAG
Trib3 R	CTCGTTTTAGGACTGGACACTTG
Irf8 F	GACACCAACCAGTTCATCCG
Irf8 R	TGCTCTACCTGCACCAGAAT
Ido1 F	TCTGCCTGTGCTGATTGAGA
Ido1 R	GCACCTTTCGAACATCGTCA
Irf1 F	ACCCTGGCTAGAGATGCAGA
Irf1 R	TGCTTTGTATCGGCCTGTGT
Mx1 F	TCTGTGCAGGCACTATGAGG
Mx1 R	GCCTCTCCACTCCTCTCCTT
