# Supplementary material for: Metabolic reprograming mediated by tumor cell-intrinsic type I IFN signaling is required for CD47-SIRPα blockade efficacy
Source: Nat Commun. 2024 Jul 9;15:5759. doi: 10.1038/s41467-024-50136-z (PMC11233683; doi:10.1038/s41467-024-50136-z)
Supplement: Supplementary file 3 — Reporting Summary [file 41467_2024_50136_MOESM3_ESM.pdf]

nature portfolio

Corresponding author(s): Mingzhao Zhu, Yang-Xin Fu, Wenjun Wang

Last updated by author(s): 06/09/2024

Reporting Summary

Nature Portfolio wishes to improve the reproducibility of the work that we publish. This form provides structure for consistency and transparency in reporting. For further information on Nature Portfolio policies, see our Editorial Policies and the Editorial Policy Checklist.

Statistics

For all statistical analyses, confirm that the following items are present in the figure legend, table legend, main text, or Methods section.

n/a	Confirmed	
☐	☒	The exact sample size (*n*) for each experimental group/condition, given as a discrete number and unit of measurement
☐	☒	A statement on whether measurements were taken from distinct samples or whether the same sample was measured repeatedly
☐	☒	The statistical test(s) used AND whether they are one- or two-sided<br>*Only common tests should be described solely by name; describe more complex techniques in the Methods section.*
☒	☐	A description of all covariates tested
☒	☐	A description of any assumptions or corrections, such as tests of normality and adjustment for multiple comparisons
☐	☒	A full description of the statistical parameters including central tendency (e.g. means) or other basic estimates (e.g. regression coefficient) AND variation (e.g. standard deviation) or associated estimates of uncertainty (e.g. confidence intervals)
☐	☒	For null hypothesis testing, the test statistic (e.g. *F*, *t*, *r*) with confidence intervals, effect sizes, degrees of freedom and *P* value noted<br>*Give P values as exact values whenever suitable.*
☒	☐	For Bayesian analysis, information on the choice of priors and Markov chain Monte Carlo settings
☒	☐	For hierarchical and complex designs, identification of the appropriate level for tests and full reporting of outcomes
☒	☐	Estimates of effect sizes (e.g. Cohen's *d*, Pearson's *r*), indicating how they were calculated

Our web collection on statistics for biologists contains articles on many of the points above.

Software and code

Policy information about availability of computer code

Data collection	Standard default instrument softwares were used for raw data collection.
Data analysis	GraphPad Prism6 was used for data analysis and plotting.<br>FlowJo was used for flow cytometry analysis.<br>ZEN2012 and ImageJ 1.51j8 software were used for immunofluoresence image analysis.<br>For RNA-seq analysis, Trim Galore was used to remove the low quality reads and adapter sequences, HISAT2 was used to align reads to the mouse genome (mm10), StringTie was used for alignments assembly analysis.

For manuscripts utilizing custom algorithms or software that are central to the research but not yet described in published literature, software must be made available to editors and reviewers. We strongly encourage code deposition in a community repository (e.g. GitHub). See the Nature Portfolio guidelines for submitting code & software for further information.

Data

Policy information about availability of data

All manuscripts must include a data availability statement. This statement should provide the following information, where applicable:

- Accession codes, unique identifiers, or web links for publicly available datasets
- A description of any restrictions on data availability
- For clinical datasets or third party data, please ensure that the statement adheres to our policy

The RNA-seq data generated in this study have been deposited in the NCBI GEO DataSets with GEO accession GSE235113. The remaining data are available within the Article, Supplementary Information or Source Data file.

Research involving human participants, their data, or biological material

Policy information about studies with human participants or human data. See also policy information about sex, gender (identity/presentation), and sexual orientation and race, ethnicity and racism.

Reporting on sex and gender	*Use the terms sex (biological attribute) and gender (shaped by social and cultural circumstances) carefully in order to avoid confusing both terms. Indicate if findings apply to only one sex or gender; describe whether sex and gender were considered in study design; whether sex and/or gender was determined based on self-reporting or assigned and methods used. Provide in the source data disaggregated sex and gender data, where this information has been collected, and if consent has been obtained for sharing of individual-level data; provide overall numbers in this Reporting Summary. Please state if this information has not been collected. Report sex- and gender-based analyses where performed, justify reasons for lack of sex- and gender-based analysis.*
Reporting on race, ethnicity, or other socially relevant groupings	*Please specify the socially constructed or socially relevant categorization variable(s) used in your manuscript and explain why they were used. Please note that such variables should not be used as proxies for other socially constructed/relevant variables (for example, race or ethnicity should not be used as a proxy for socioeconomic status). Provide clear definitions of the relevant terms used, how they were provided (by the participants/respondents, the researchers, or third parties), and the method(s) used to classify people into the different categories (e.g. self-report, census or administrative data, social media data, etc.) Please provide details about how you controlled for confounding variables in your analyses.*
Population characteristics	*Describe the covariate-relevant population characteristics of the human research participants (e.g. age, genotypic information, past and current diagnosis and treatment categories). If you filled out the behavioural & social sciences study design questions and have nothing to add here, write "See above."*
Recruitment	*Describe how participants were recruited. Outline any potential self-selection bias or other biases that may be present and how these are likely to impact results.*
Ethics oversight	*Identify the organization(s) that approved the study protocol.*

Note that full information on the approval of the study protocol must also be provided in the manuscript.

Field-specific reporting

Please select the one below that is the best fit for your research. If you are not sure, read the appropriate sections before making your selection.

☒ Life sciences ☐ Behavioural & social sciences ☐ Ecological, evolutionary & environmental sciences

For a reference copy of the document with all sections, see nature.com/documents/nr-reporting-summary-flat.pdf

Life sciences study design

All studies must disclose on these points even when the disclosure is negative.

Sample size	We did not predetermine sample size using software. The exact n values used to calculate the statistics are provided and a reasonable sample size was chosen to ensure adequate reproducibility of results.
Data exclusions	No data were excluded from the analysis.
Replication	All experiments were performed at least twice with similar results.
Randomization	Mice were randomized into different groups.
Blinding	Blinding was not performed due to the unambiguous nature of measurements and systematic analyses used in these experiments.

Reporting for specific materials, systems and methods

We require information from authors about some types of materials, experimental systems and methods used in many studies. Here, indicate whether each material, system or method listed is relevant to your study. If you are not sure if a list item applies to your research, read the appropriate section before selecting a response.

Materials & experimental systems

n/a	Involved in the study
☐	☒ Antibodies
☐	☒ Eukaryotic cell lines
☒	☐ Palaeontology and archaeology
☐	☒ Animals and other organisms
☒	☐ Clinical data
☒	☐ Dual use research of concern
☒	☐ Plants

Methods

n/a	Involved in the study
☒	☐ ChIP-seq
☐	☒ Flow cytometry
☒	☐ MRI-based neuroimaging

Antibodies

Antibodies used

CD45 PerCP-5.5(Ly-5) (Clone: 30-F11; Cat. 45-0451; eBioscience)
CD3e FITC (Clone: 145-2C11; Cat. 11-0031-82; eBioscience)
CD19 PE (Clone: eBio1D3 (1D3); Cat. 12-0193-82; eBioscience)
CD11c APC (Clone: N418; Cat. 117310; Biolegend)
I-A/I-E (MHCII) APC/Cy7 (Clone: M5/114.15.2; Cat. 107628; Biolegend)
CD45.1 PE/Cy7 (Clone: A20; Cat. 25-0453-81; eBioscience)
CD44 APC/Cy7 (Clone: IM7; Cat. 103028; Biolegend)
CD4 PE/Cy7 (Clone:GK1.5; Cat. 25-0041-82; eBioscience)
CD8 APC-eFlourR 780 (Clone: 53-6.7; Cat. 47-0081-82; eBioscience)
Foxp3 APC (Clone: FJK-16s; Cat. 17-5773-82; eBioscience)
CD80 PE (Clone:16-10A1; Cat. 12-0801; Lot: E01357-234; eBioscience)
CD39 PE (Clone:Duha59; Cat. 143803; Lot: B279087;Biolegend)
CD73 biotin (Clone: TY/11.8; Cat. 130-102-046; Miltenyi)
CD47 APC (Clone: miap301; Cat. 17-0471-80; eBioscience)
IFNγPE (Clone: XMG1.2; Cat. 505808; Biolegend)
TCRVα2 FITC (Clone: B20.1; Cat. 11-5812-82; eBioscience)
CD69 PE (Clone: H1.2F3; Cat. 12-0691; eBioscience)
CD62L Alexa Fluor® 700 (Clone: MEL-14; Cat. 56-0621-82; eBioscience)
IFNAR-1 PE (Clone: MAR1-5A3; Cat. 127311; Lot: B227012; Biolegend)
CD119 (IFN gamma Receptor 1) PE (Clone: 2E2; Cat. 12-1191-80; eBioscience)
Phospho-STAT1 (Ser727) Polyclonal Antibody (Cat. 28977-1-AP; Proteintech)
IRF1 Polyclonal Antibody (Cat. 11335-1-AP; Proteintech)
LC3 (Cat. L8918; Sigma-Aldrich)
β-actin (Clone: AC-15; Cat. A3854; Sigma-Aldrich)
GAPDH (Cat. SAB1410512; Sigma-Aldrich)
Purified Anti-Mouse IFN-γ (Cat. 51-2525KC; BD Biosciences)
Biotinylated anti-mouse IFN-γ (Cat. 51-1818KZ; BD Biosciences)
streptavidin-HRP (Cat. 51-9000209; BD Biosciences)

Validation

CD45 PerCP-5.5(Ly-5) (Clone: 30-F11; Cat. 45-0451; eBioscience)
https://www.thermofisher.cn/cn/zh/antibody/product/CD45-Antibody-clone-30-F11-Monoclonal/45-0451-82
CD3e FITC (Clone: 145-2C11; Cat. 11-0031-82; eBioscience)
https://www.thermofisher.cn/cn/zh/antibody/product/CD3e-Antibody-clone-145-2C11-Monoclonal/11-0031-82
CD19 PE (Clone: eBio1D3 (1D3); Cat. 12-0193-82; eBioscience)
https://www.thermofisher.cn/cn/zh/antibody/product/CD19-Antibody-clone-eBio1D3-1D3-Monoclonal/12-0193-82
CD11c APC (Clone: N418; Cat. 117310; Biolegend)
https://www.biolegend.com/en-us/products/apc-anti-mouse-cd11c-antibody-1813
I-A/I-E (MHCII) APC/Cy7 (Clone: M5/114.15.2; Cat. 107628; Biolegend)
https://www.biolegend.com/en-us/products/apc-cyanine7-anti-mouse-i-a-i-e-antibody-5966
CD45.1 PE/Cy7 (Clone: A20; Cat. 25-0453-81; eBioscience)
https://www.thermofisher.cn/cn/zh/antibody/product/CD45-1-Antibody-clone-A20-Monoclonal/25-0453-81
CD44 APC/Cy7 (Clone: IM7; Cat. 103028; Biolegend)
https://www.biolegend.com/en-us/products/apc-cyanine7-anti-mouse-human-cd44-antibody-3933
CD4 PE/Cy7 (Clone:GK1.5; Cat. 25-0041-82; eBioscience)
https://www.thermofisher.cn/cn/zh/antibody/product/CD4-Antibody-clone-GK1-5-Monoclonal/25-0041-82
CD8 APC-eFlourR 780 (Clone: 53-6.7; Cat. 47-0081-82; eBioscience)

https://www.thermofisher.cn/cn/zh/antibody/product/CD8a-Antibody-clone-53-6-7-Monoclonal/47-0081-82
Foxp3 APC (Clone: FJK-16s; Cat. 17-5773-82; eBioscience)
https://www.thermofisher.cn/cn/zh/antibody/product/FOXP3-Antibody-clone-FJK-16s-Monoclonal/17-5773-82
CD80 PE (Clone:16-10A1; Cat. 12-0801; Lot: E01357-234; eBioscience)
https://www.thermofisher.cn/cn/zh/antibody/product/CD80-B7-1-Antibody-clone-16-10A1-Monoclonal/12-0801-83
CD39 PE (Clone:Duha59; Cat. 143803; Lot: B279087;Biolegend)
https://www.biolegend.com/en-gb/products/pe-anti-mouse-cd39-antibody-7812
CD73 biotin (Clone: TY/11.8; Cat. 130-102-046; Miltenyi)
https://www.miltenyibiotec.com/CN-en/products/cd73-antibody-anti-mouse-ty-11-8.html#conjugate=biotin:size=9-ug-in-300-ul
CD47 APC (Clone: miap301; Cat. 17-0471-80; eBioscience)
https://www.thermofisher.cn/cn/zh/antibody/product/CD47-Antibody-clone-miap301-Monoclonal/17-0471-80
IFNγPE (Clone: XMG1.2; Cat. 505808; Biolegend)
https://www.biolegend.com/en-gb/products/pe-anti-mouse-ifn-gamma-antibody-997
TCRVα2 FITC (Clone: B20.1; Cat. 11-5812-82; eBioscience)
https://www.thermofisher.cn/cn/zh/antibody/product/TCR-V-alpha-2-Antibody-clone-B20-1-Monoclonal/11-5812-82
CD69 PE (Clone: H1.2F3; Cat. 12-0691; eBioscience)
https://www.thermofisher.cn/cn/zh/antibody/product/CD69-Antibody-clone-H1-2F3-Monoclonal/12-0691-81
CD62L Alexa Fluor® 700 (Clone: MEL-14; Cat. 56-0621-82; eBioscience)
https://www.thermofisher.cn/cn/zh/antibody/product/CD62L-L-Selectin-Antibody-clone-MEL-14-Monoclonal/56-0621-82
IFNAR-1 PE (Clone: MAR1-5A3; Cat. 127311; Lot: B227012; Biolegend)
https://www.biolegend.com/en-gb/products/pe-anti-mouse-ifnar-1-antibody-4784
CD119 (IFN gamma Receptor 1) PE (Clone: 2E2; Cat. 12-1191-80; eBioscience)
https://www.thermofisher.cn/cn/zh/antibody/product/CD119-IFN-gamma-Receptor-1-Antibody-clone-2E2-Monoclonal/12-1191-80
Phospho-STAT1 (Ser727) Polyclonal Antibody (Cat. 28977-1-AP; Proteintech)
https://www.ptgcn.com/products/Phospho-STAT1-Ser727-Antibody-28977-1-AP.htm
IRF1 Polyclonal Antibody (Cat. 11335-1-AP; Proteintech)
https://www.ptgcn.com/products/IRF1-Antibody-11335-1-AP.htm
LC3 (Cat. L8918; Sigma-Aldrich)
https://www.sigmaaldrich.cn/CN/zh/product/sigma/l8918
β-actin (Clone: AC-15; Cat. A3854; Sigma-Aldrich)
https://www.sigmaaldrich.cn/CN/zh/product/sigma/a3854
GAPDH (Cat. SAB1410512; Sigma-Aldrich)
https://www.sigmaaldrich.cn/CN/zh/product/sigma/sab1410512
Purified Anti-Mouse IFN-γ (Cat. 51-2525KC; BD Biosciences)
Biotinylated anti-mouse IFN-γ (Cat. 51-1818KZ; BD Biosciences)
streptavidin-HRP (Cat. 51-9000209; BD Biosciences)
https://www.bdbiosciences.com/zh-cn/products/reagents/immunoassay-reagents/elispot/elispot-kits/mouse-ifng-elispot-set.551083

Eukaryotic cell lines

Policy information about cell lines and Sex and Gender in Research

Cell line source(s)	MC38, CT26, A20 and HT29 cells were from ATCC. IFNAR1, IFNGAR, Atg5, Ndufs6 and Isg15 deficient tumor cells were generated using CRISPR/Cas9 technology.
Authentication	No authentication was carried out. Cell morphology and adhesion was consistent with expectations.
Mycoplasma contamination	Cell lines were tested to be negative for Mycoplasma.
Commonly misidentified lines (See ICLAC register)	No commonly misidentified cell lines were used in this study.

Animals and other research organisms

Policy information about studies involving animals; ARRIVE guidelines recommended for reporting animal research, and Sex and Gender in Research

Laboratory animals	WT C57BL/6 and BALB/c mice were purchased from Vital River, a Charles River company in China. P2X7R KO mice were provided by Prof. Jinhui Tao (University of Science and Technology of China, Hefei, China). Tcra-/- and OT-I TCR transgenic mice were purchased from The Jackson Laboratory.
Wild animals	No wild animals were involved.
Reporting on sex	Sex-associated phenotypes or mechanisms were not the focus for designing experiments in our study.The sex of animals used in the study was female.
Field-collected samples	No field-collected samples were used.

Ethics oversight	All mice were maintained under specific pathogen-free conditions and all animal experimental procedures were performed with approval (SYXK2020035) from the institutional committee of the Institute of Biophysics, Chinese Academy of Sciences.

Note that full information on the approval of the study protocol must also be provided in the manuscript.

Flow Cytometry

Plots

Confirm that:

- [x] The axis labels state the marker and fluorochrome used (e.g. CD4-FITC).
- [x] The axis scales are clearly visible. Include numbers along axes only for bottom left plot of group (a 'group' is an analysis of identical markers).
- [x] All plots are contour plots with outliers or pseudocolor plots.
- [x] A numerical value for number of cells or percentage (with statistics) is provided.

Methodology

Sample preparation	Tumors and draining lymph nodes were isolated from MC38-bearing mice and digested into single-cell suspensions using collagenase I (1 mg/mL) and DNase (500 U/mL) at 37°C for 1 h.
Instrument	LSRFortessa, FACS Aria III
Software	BD FACS Diva 8.0.1 software was used for data collection and FlowJo v.10 was used for data analysis.
Cell population abundance	The percentage of different subpopulation of cells was analyzed using the gating strategy as described below.
Gating strategy	Apoptotic cells were stained with annexin V and 7-AAD, and double positive population was gated.

- [x] Tick this box to confirm that a figure exemplifying the gating strategy is provided in the Supplementary Information.
