# Supplementary material for: Metabolic reprograming mediated by tumor cell-intrinsic type I IFN signaling is required for CD47-SIRPα blockade efficacy
Source: Nat Commun. 2024 Jul 9;15:5759. doi: 10.1038/s41467-024-50136-z (PMC11233683; doi:10.1038/s41467-024-50136-z)
Supplement: Supplementary file 4 — Source Data [file 41467_2024_50136_MOESM4_ESM.zip › Source data/Unedited blot and gel images 20240626.pptx]

## Slide 1
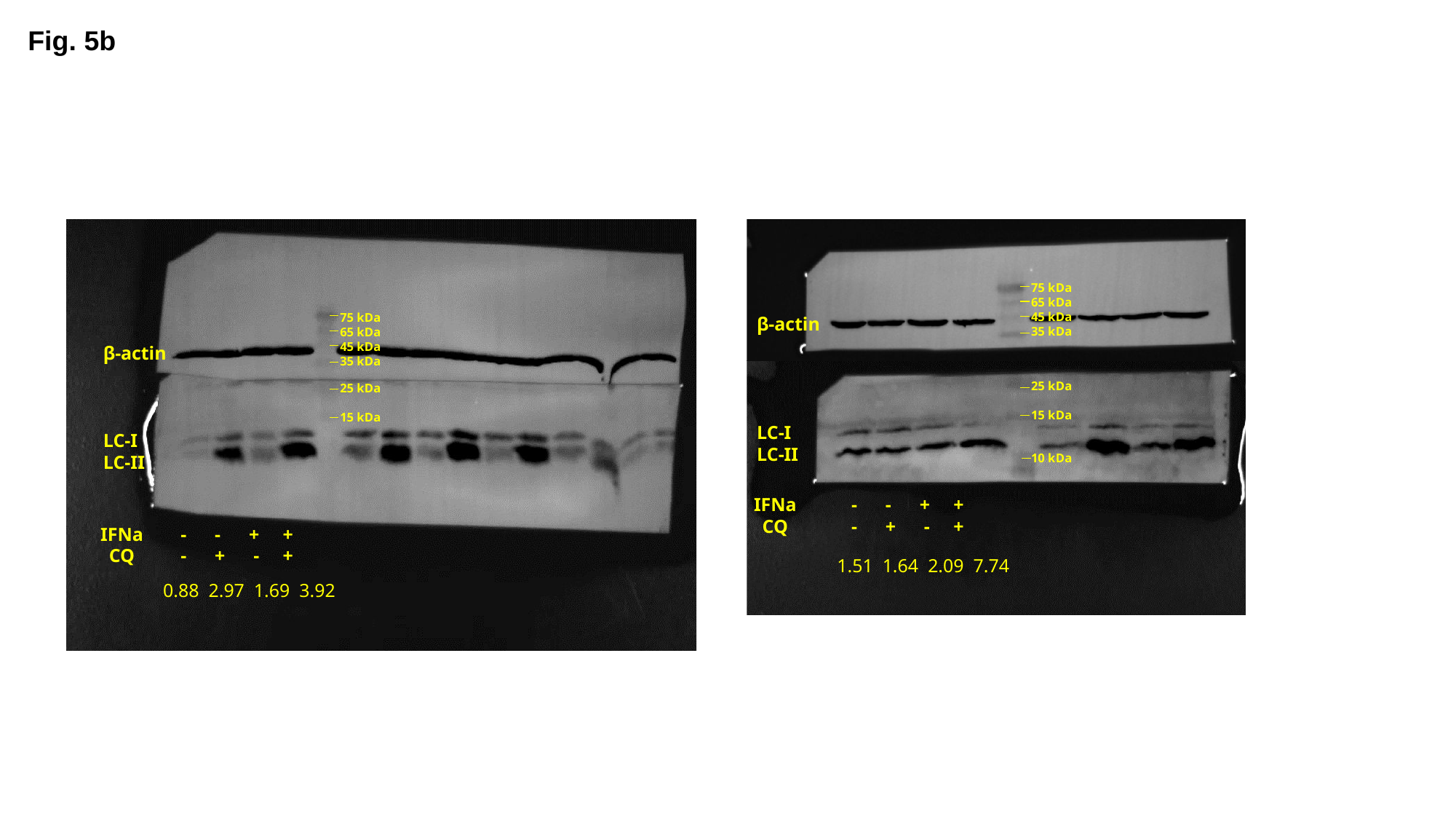

Fig. 5b
75 kDa
65 kDa
45 kDa
35 kDa
25 kDa
15 kDa
10 kDa
β-actin
LC-I
LC-II
75 kDa
65 kDa
45 kDa
35 kDa
25 kDa
15 kDa
β-actin
LC-I
LC-II
IFNa
CQ
 - - + +
 - + - +
IFNa
CQ
 - - + +
 - + - +
 1.51 1.64 2.09 7.74
0.88 2.97 1.69 3.92

## Slide 2
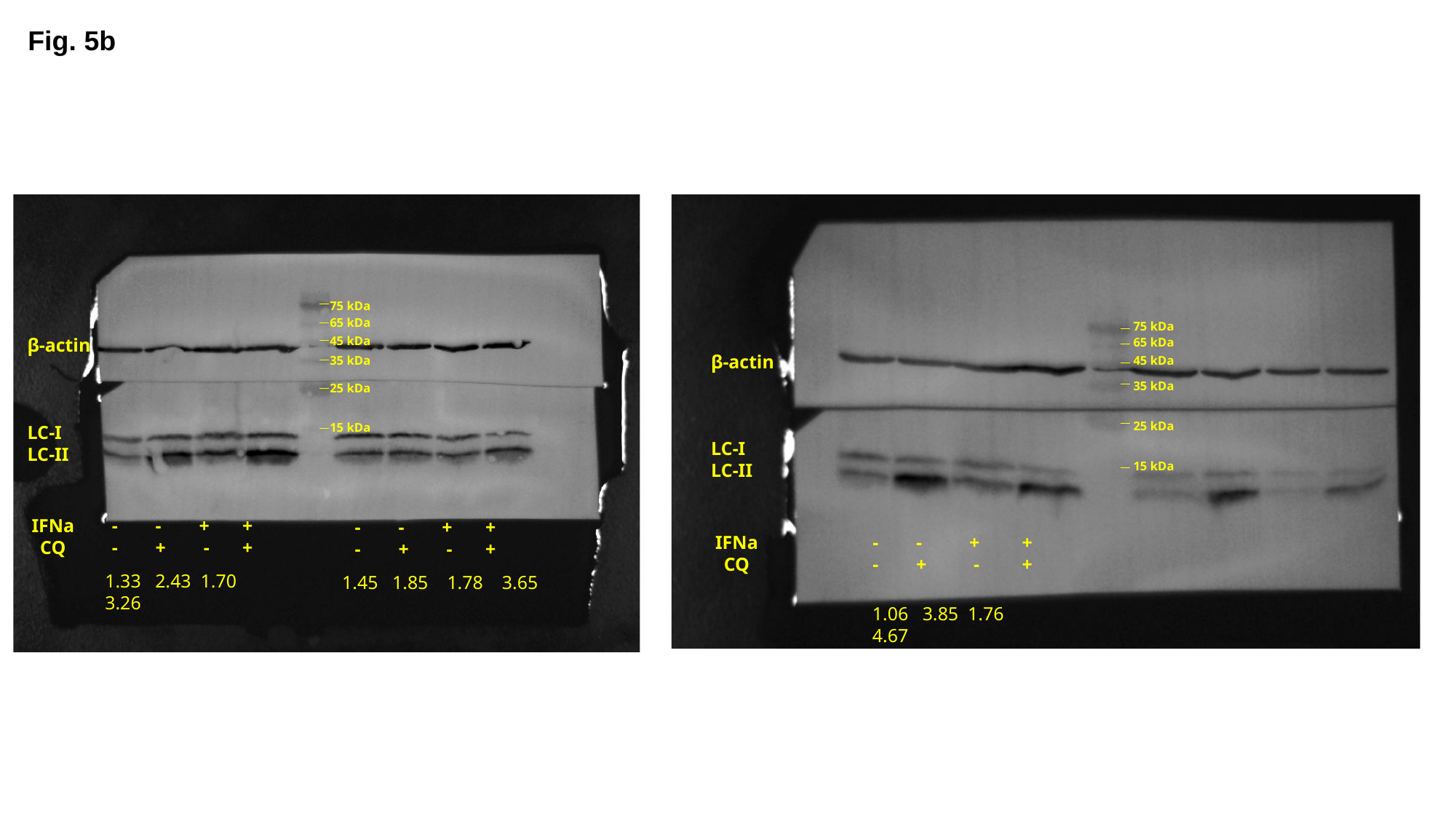

Fig. 5b
75 kDa
65 kDa
45 kDa
35 kDa
25 kDa
15 kDa
75 kDa
65 kDa
45 kDa
35 kDa
25 kDa
15 kDa
β-actin
LC-I
LC-II
β-actin
LC-I
LC-II
IFNa
CQ
 - - + +
 - + - +
 - - + +
 - + - +
IFNa
CQ
 - - + +
 - + - +
1.33 2.43 1.70 3.26
1.45 1.85 1.78 3.65
1.06 3.85 1.76 4.67

## Slide 3
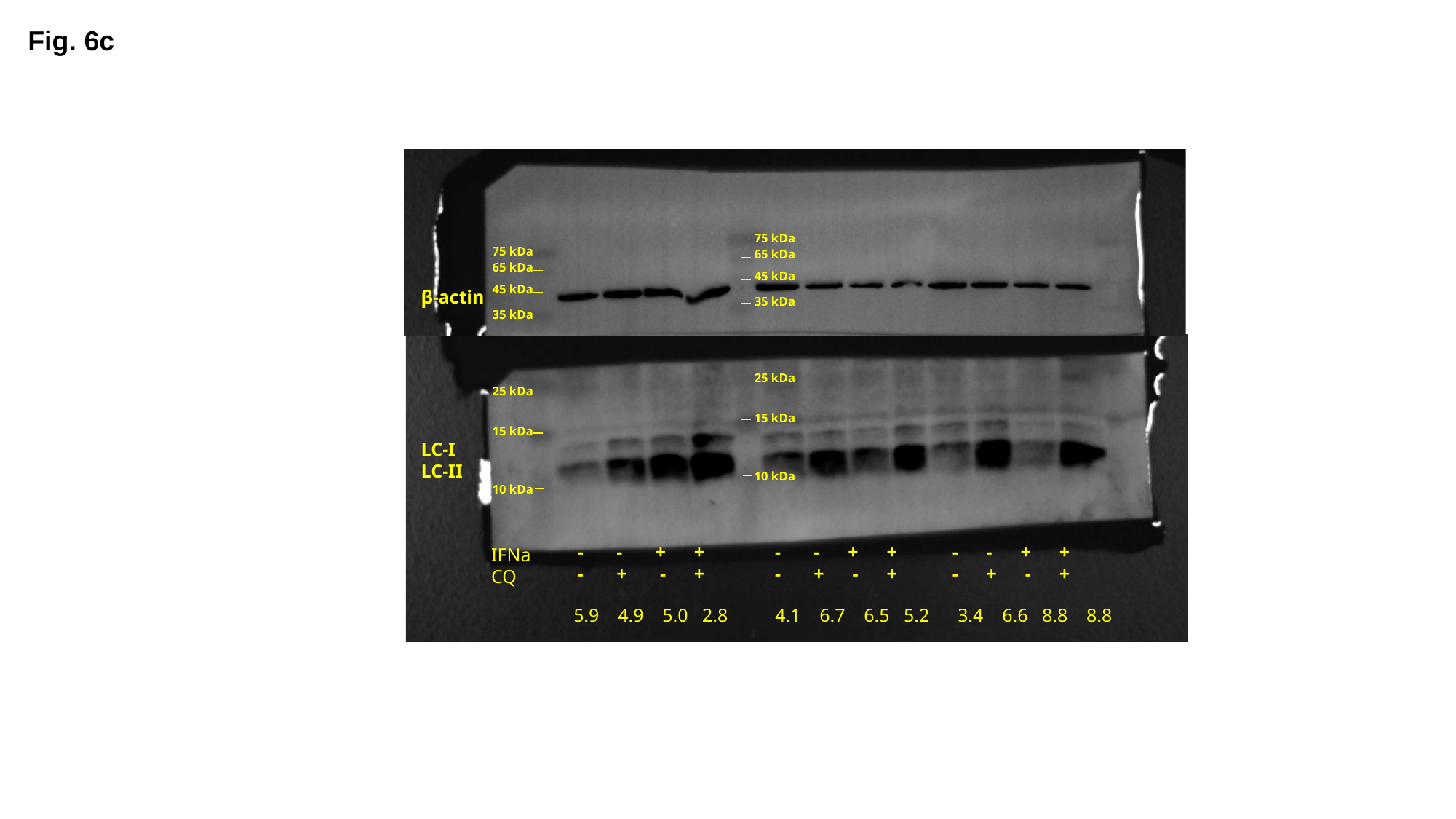

Fig. 6c
75 kDa
65 kDa
45 kDa
35 kDa
25 kDa
15 kDa
10 kDa
75 kDa
65 kDa
45 kDa
35 kDa
25 kDa
15 kDa
10 kDa
β-actin
LC-I
LC-II
 - - + +
 - + - +
 - - + +
 - + - +
 - - + +
 - + - +
IFNa
CQ
5.9 4.9 5.0 2.8 4.1 6.7 6.5 5.2 3.4 6.6 8.8 8.8

## Slide 4
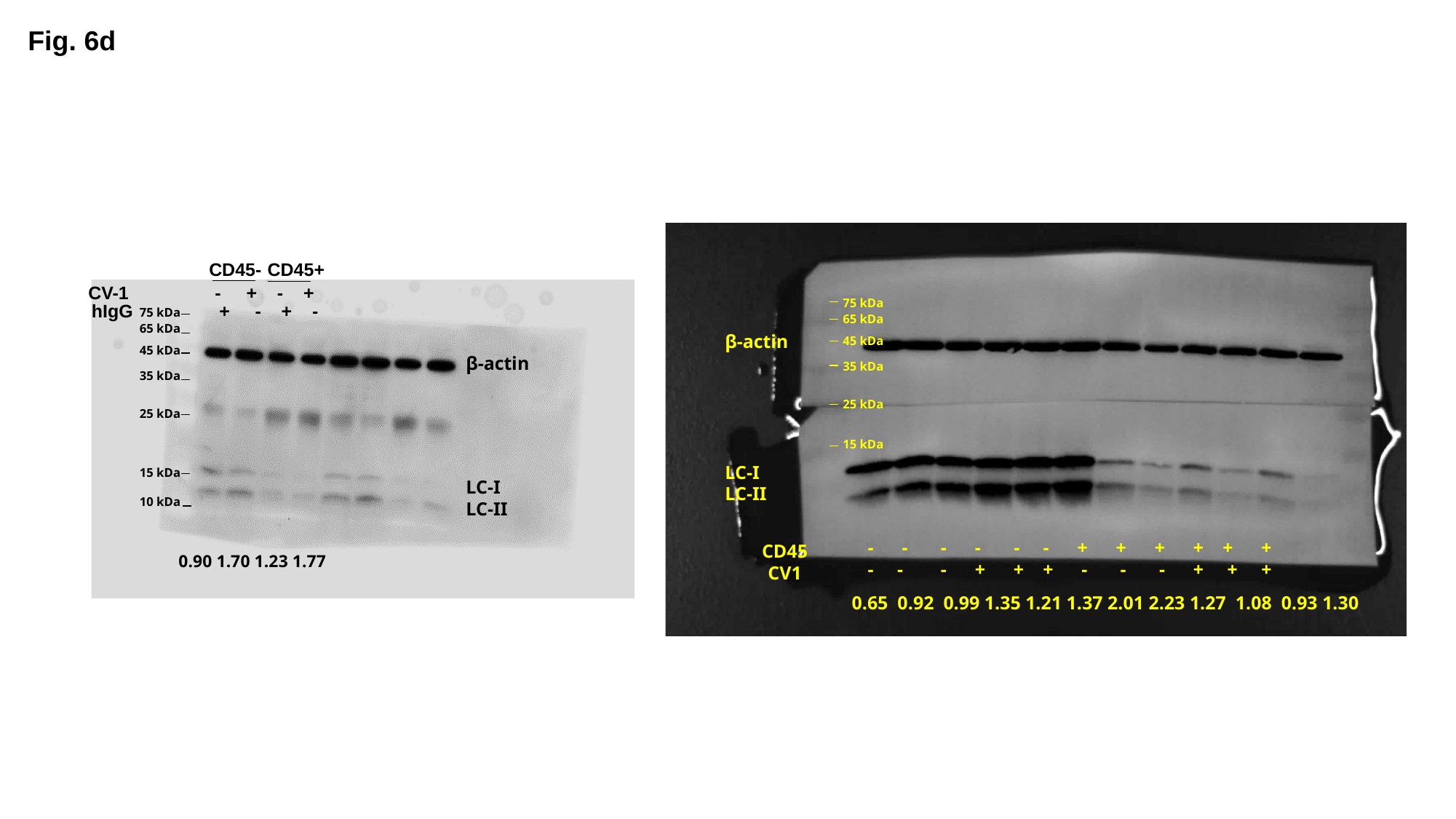

Fig. 6d
CD45-
CD45+
75 kDa
65 kDa
45 kDa
35 kDa
25 kDa
15 kDa
CV-1 - + - +
75 kDa
65 kDa
45 kDa
35 kDa
25 kDa
15 kDa
10 kDa
hIgG + - + -
β-actin
LC-I
LC-II
β-actin
LC-I
LC-II
 - - - - - - + + + + + +
 - - - + + + - - - + + +
CD45
CV1
0.90 1.70 1.23 1.77
0.65 0.92 0.99 1.35 1.21 1.37 2.01 2.23 1.27 1.08 0.93 1.30

## Slide 5
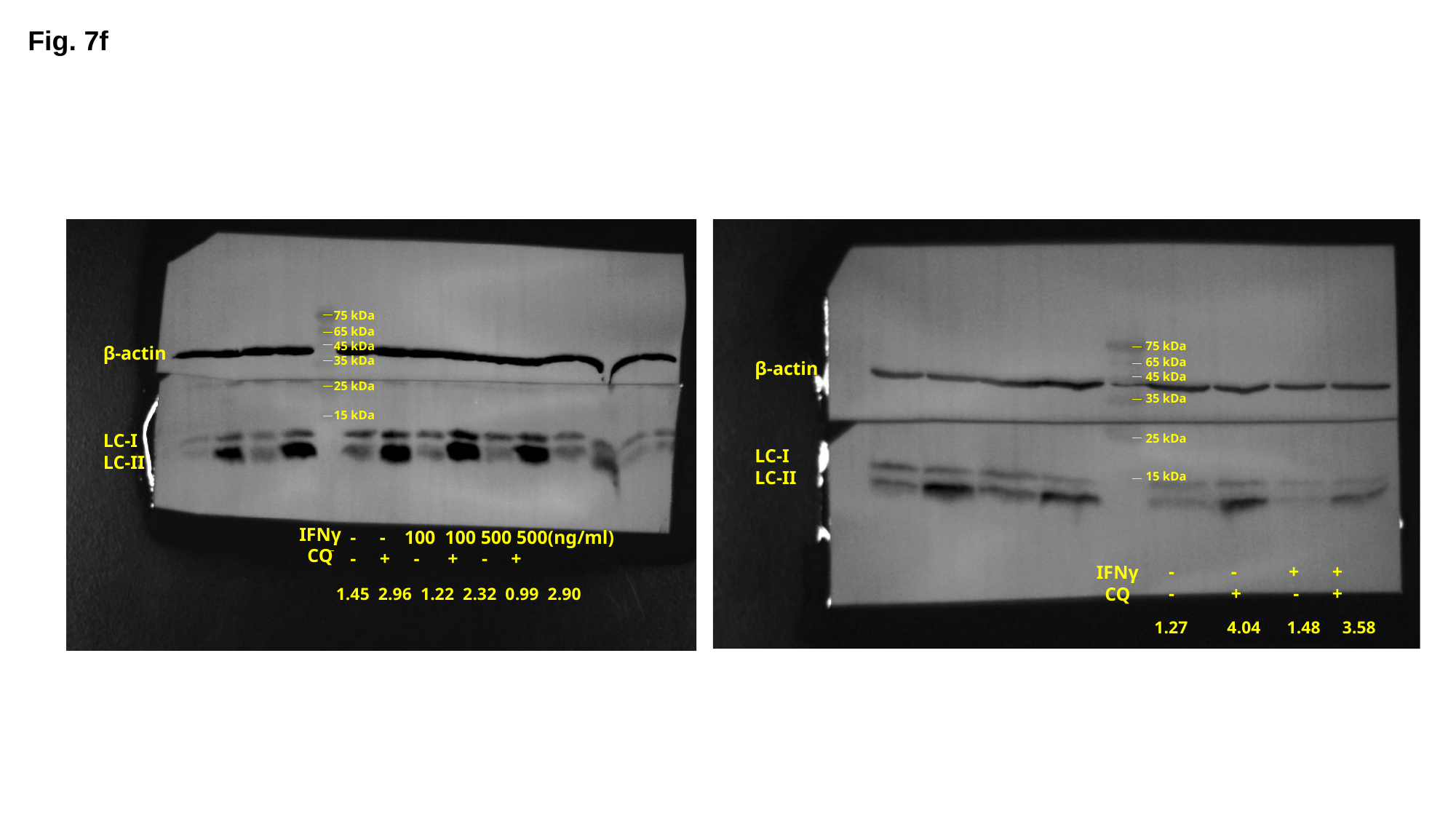

Fig. 7f
75 kDa
65 kDa
45 kDa
35 kDa
25 kDa
15 kDa
β-actin
LC-I
LC-II
75 kDa
65 kDa
45 kDa
35 kDa
25 kDa
15 kDa
β-actin
LC-I
LC-II
IFNγ
CQ
 - - 100 100 500 500(ng/ml)
 - + - + - +
 - - + +
 - + - +
IFNγ
CQ
1.45 2.96 1.22 2.32 0.99 2.90
1.27 4.04 1.48 3.58

## Slide 6
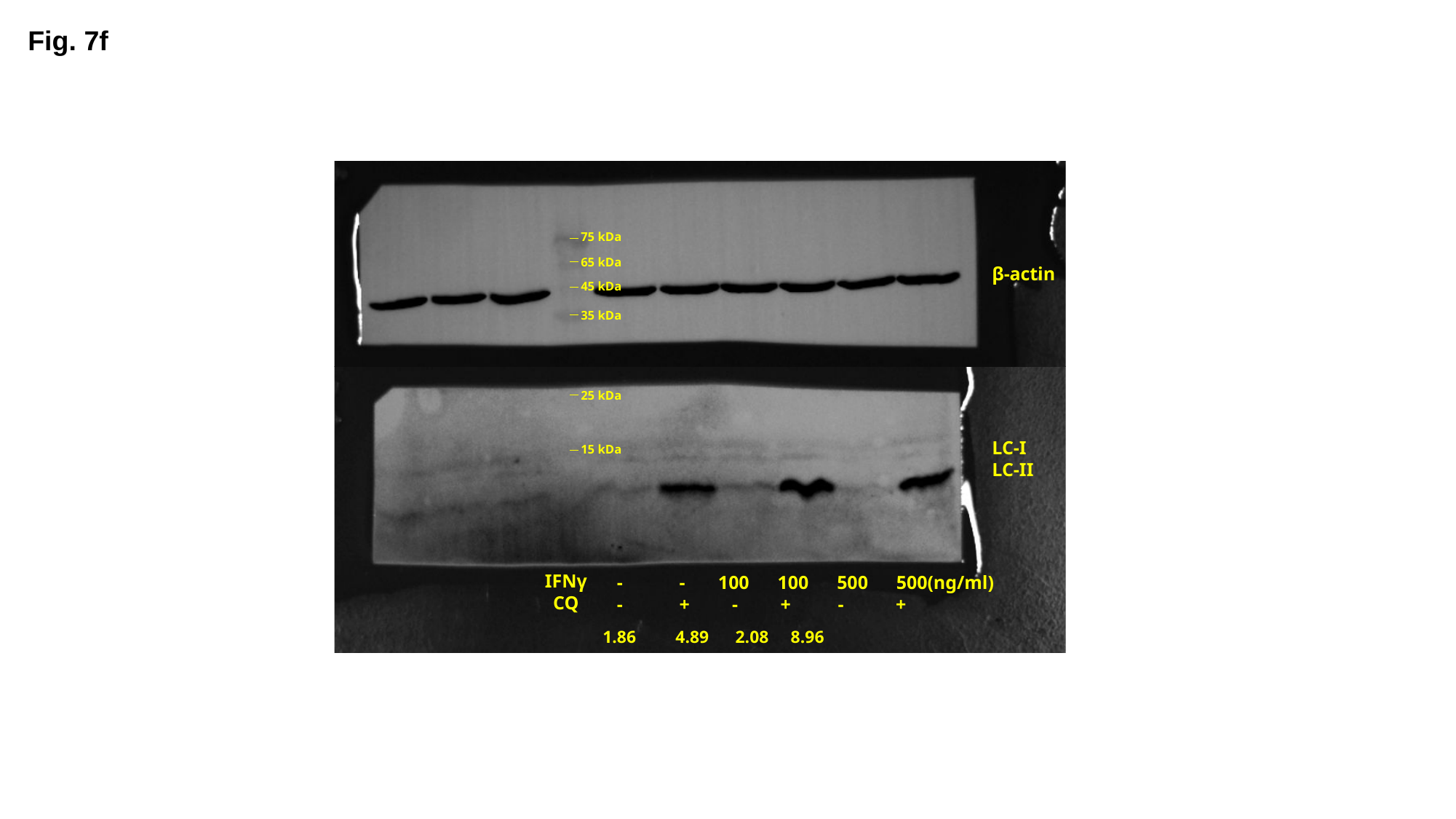

Fig. 7f
75 kDa
65 kDa
45 kDa
35 kDa
25 kDa
15 kDa
β-actin
LC-I
LC-II
IFNγ
CQ
 - - 100 100 500 500(ng/ml)
 - + - + - +
1.86 4.89 2.08 8.96

## Slide 7
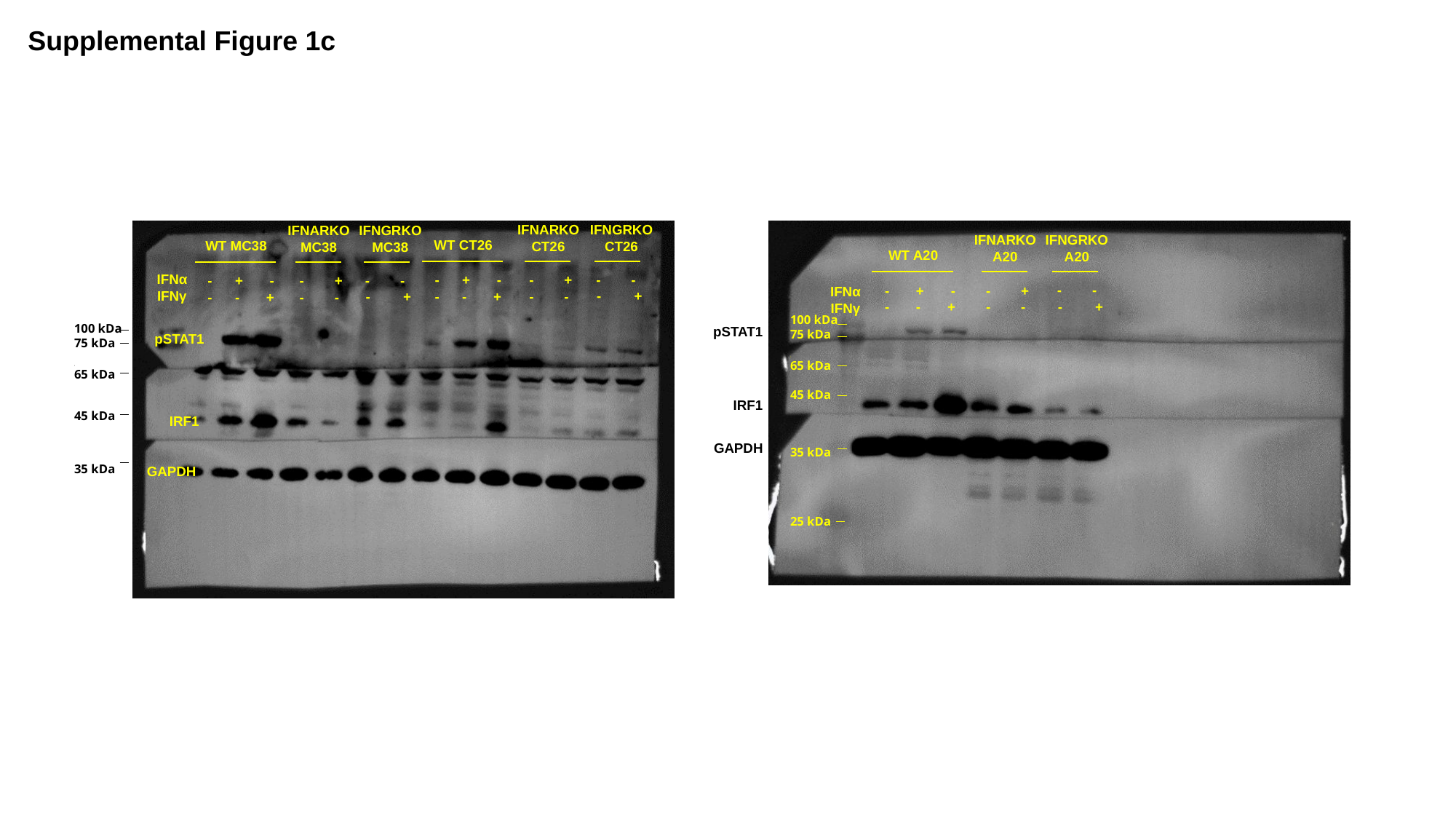

Supplemental Figure 1c
IFNARKO CT26
IFNGRKO CT26
IFNARKO MC38
IFNGRKO MC38
IFNARKO A20
IFNGRKO A20
WT CT26
WT MC38
WT A20
IFNα
IFNγ
 - -
 - +
 - + -
 - - +
 - +
 - -
 - -
 - +
 - + -
 - - +
 - +
 - -
 - -
 - +
 - + -
 - - +
 - +
 - -
IFNα
IFNγ
100 kDa
75 kDa
\
65 kDa
45 kDa
35 kDa
25 kDa
100 kDa
75 kDa
\
65 kDa
45 kDa
35 kDa
pSTAT1
pSTAT1
IRF1
IRF1
GAPDH
GAPDH

## Slide 8
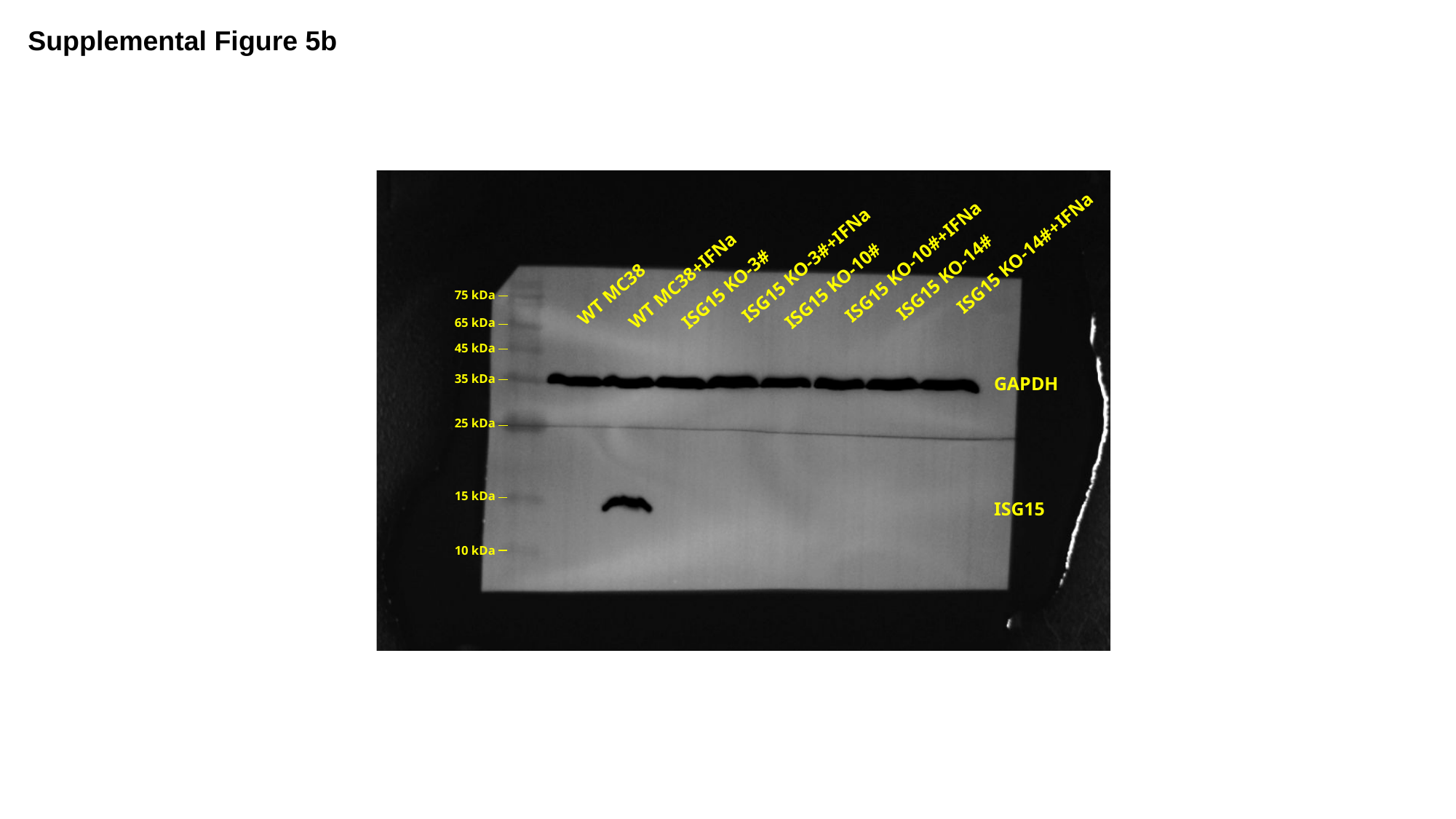

Supplemental Figure 5b
ISG15 KO-14#+IFNa
ISG15 KO-10#+IFNa
ISG15 KO-3#+IFNa
WT MC38+IFNa
ISG15 KO-14#
ISG15 KO-3#
ISG15 KO-10#
75 kDa
65 kDa
45 kDa
35 kDa
25 kDa
15 kDa
10 kDa
WT MC38
GAPDH
ISG15

## Slide 9
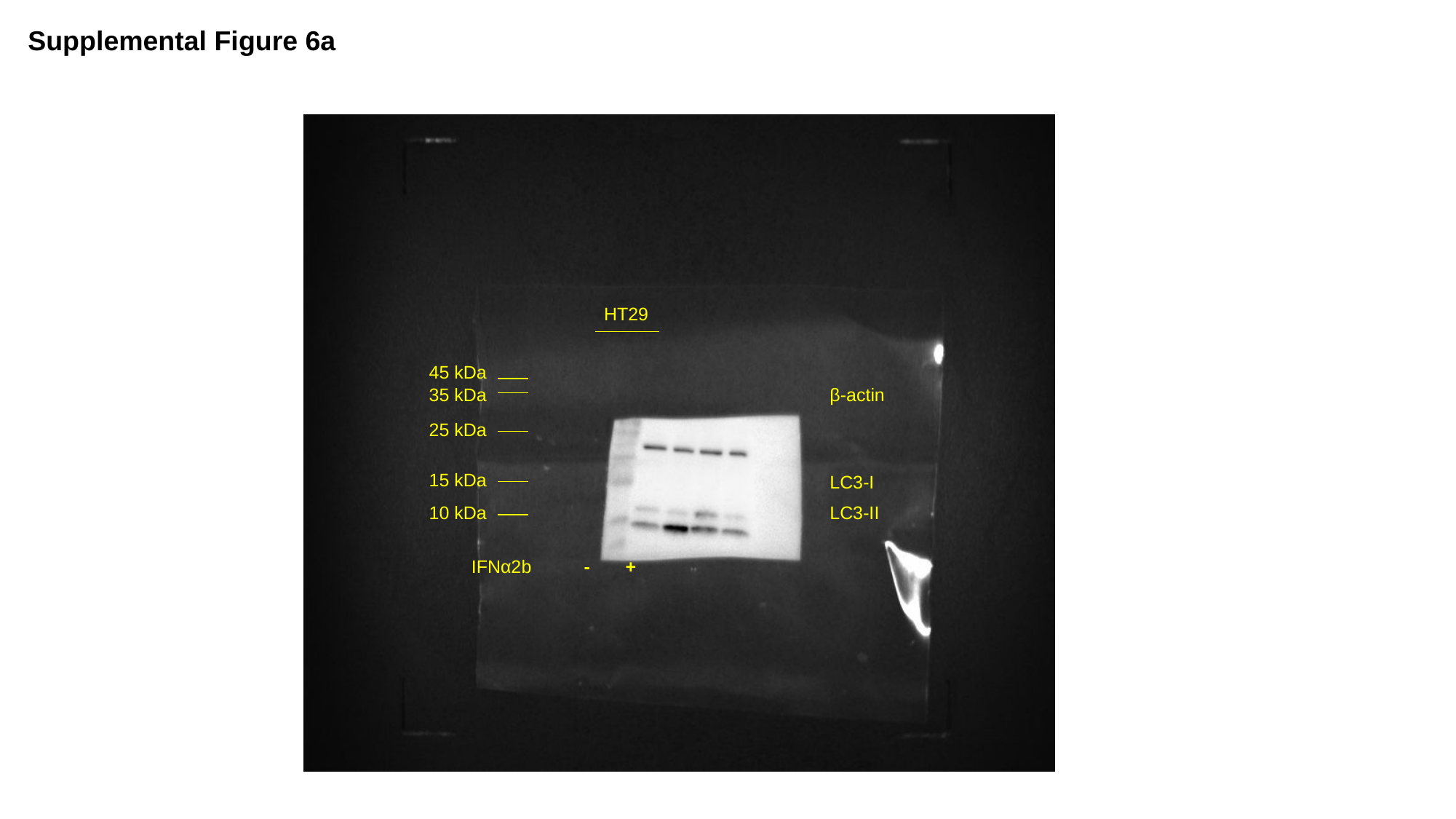

Supplemental Figure 6a
HT29
45 kDa
35 kDa
β-actin
25 kDa
15 kDa
LC3-I
LC3-II
10 kDa
- +
IFNα2b

## Slide 10
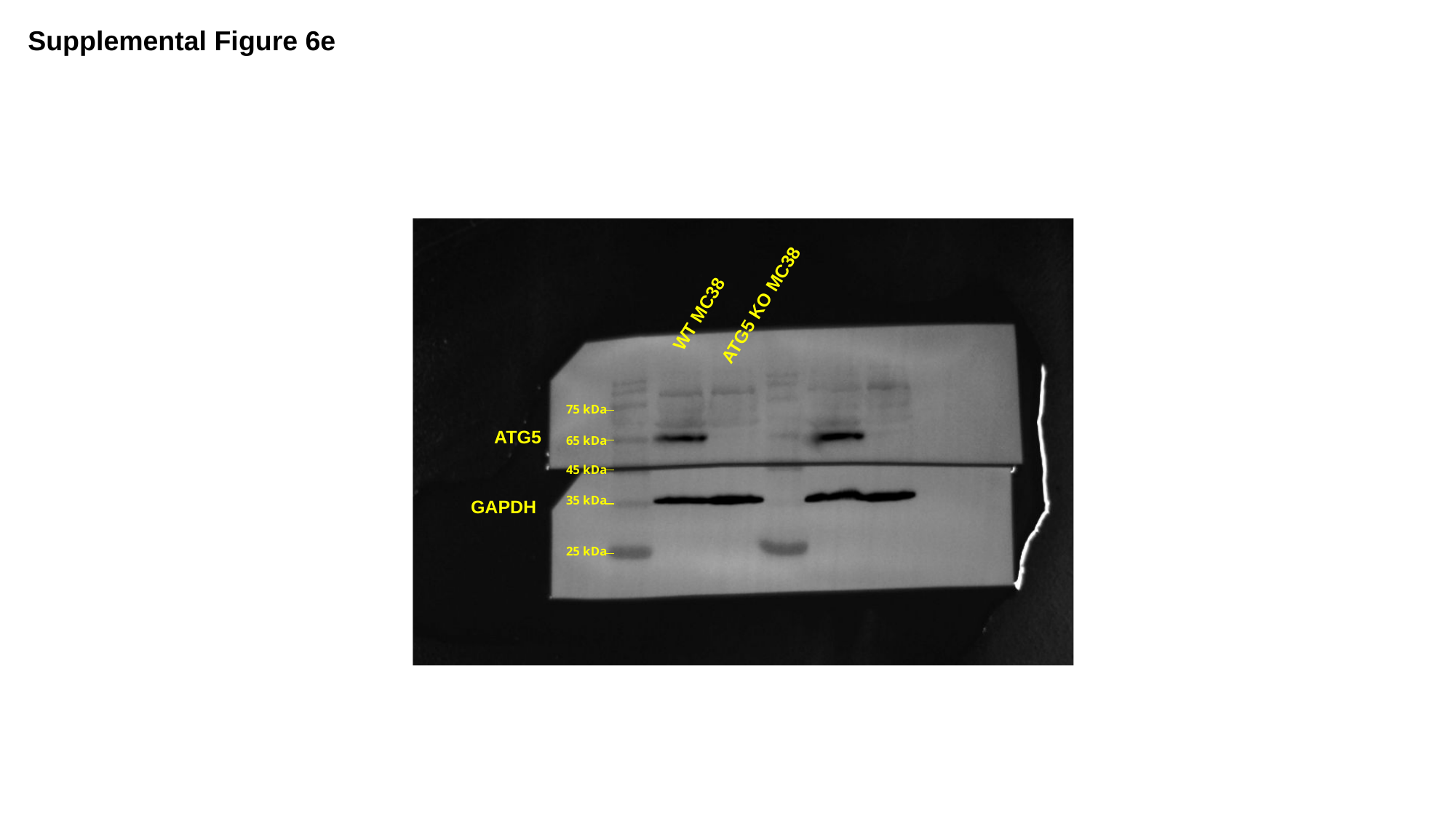

Supplemental Figure 6e
ATG5 KO MC38
WT MC38
75 kDa
65 kDa
45 kDa
35 kDa
25 kDa
ATG5
GAPDH

## Slide 11
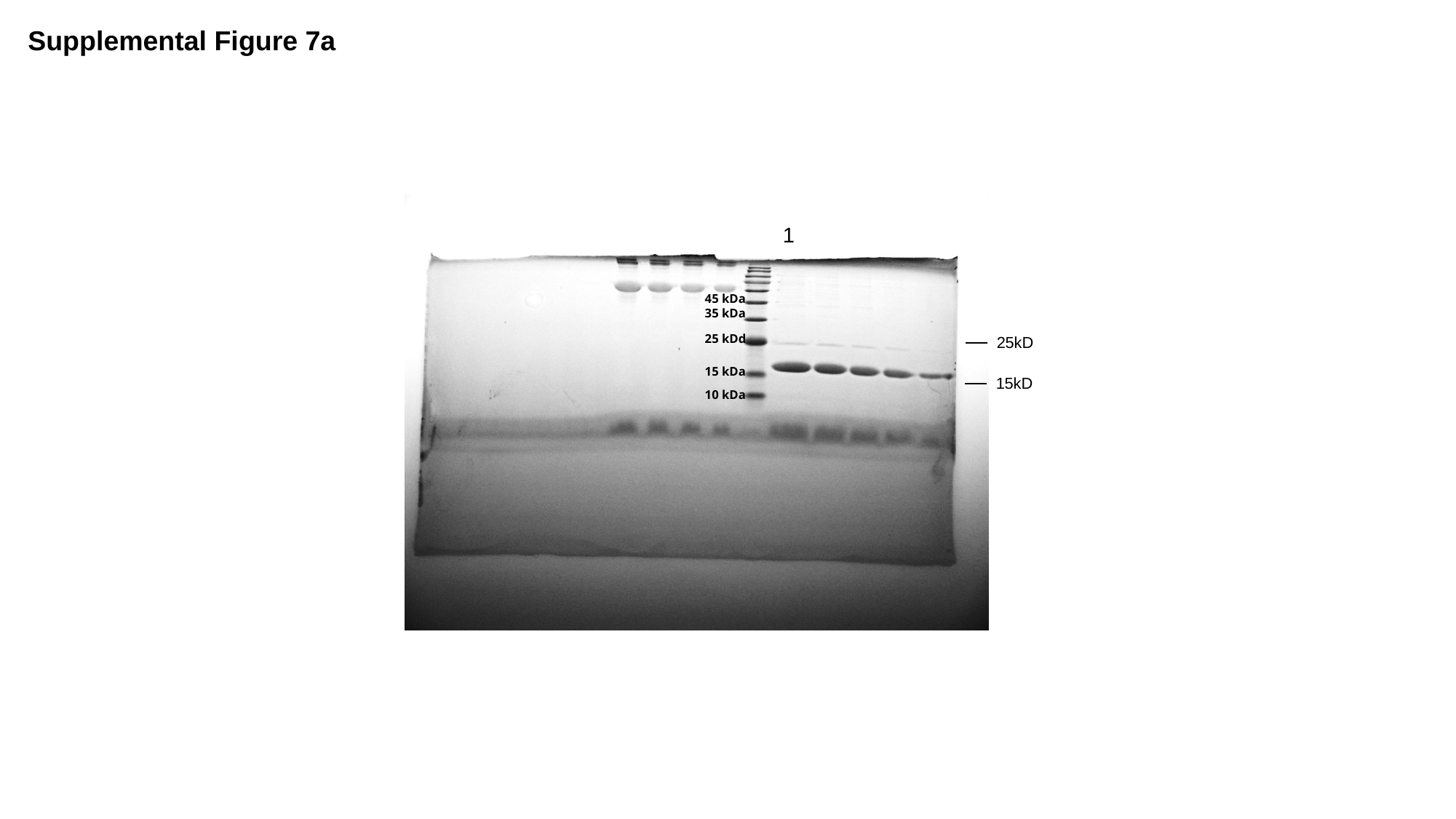

Supplemental Figure 7a
1
45 kDa
35 kDa
25 kDd
15 kDa
10 kDa
25kD
15kD
